# Medical Opinion and Movement

**Published:** 1907-11-16

**Authors:** 


### Medical Opinion and Movement.

In a recent action tried before Mr. Justice Darling a boy was awarded £750 damages for serious injury sustained through being knocked down by an omnibus. On the question being raised by counsel as to the disposal of the sum, Mr. Justice Darling expressed the opinion that a substantial contribution from it should be made to the hospital where he had received such skilled surgical treatment, but for which the boy would have died. The jury fully accorded with the sentiments of the judge. It certainly seems only just that in such circumstances the hospital should receive some recognition from the individual when he is in a position to make it, though this is probably the first time that such a right has been recognised by a judge. Sir Edward Ridley has since publicly expressed his entire approval of the course adopted by Sir Charles Darling, in a letter to the *Times*, and he suggests that in suitable cases the court should have power to allot a definite sum to a hospital from the damages assessed, as remuneration for services rendered.

Some years ago Dr. Cordier, of Lyons, recommended the subcutaneous injection of air as a therapeutic measure for the relief of pain, especially in cases of neuralgia and in sciatica. The subject has recently been taken up by Dr. Alfred S. Gubb, of Aix-les-Bains and Algiers, and he advocates this method of treatment in all cases of neuralgia and neuritis and in cases of neuralgic pain following Herpes zona. The technique is quite simple. The apparatus consists of an ordinary rubber bulb provided with an elastic reservoir such as is used for Paquelin's cautery. This is connected with a hypodermic needle by rubber tubing, in which is inserted a glass bulb filled with sterilised cotton wool, in order to free the air from all impurities. The needle must, of course, be sterilised, and also the skin of the part to be treated. The needle is inserted under the skin, and after waiting to see that no blood exudes, and that it has not, therefore, entered a blood-vessel, insufflation is commenced. The injected air causes rounded swellings which rapidly spread in the course of a vascular or nervous sheath. The skin is first blanched and then becomes reddened. The characteristic crepitation of surgical emphysema is produced. There is no pain even when comparatively large volumes of air are injected, but cutaneous sensibility is diminished and a feeling of numbness results. The amount of air introduced varies according to the region. Over the thorax 10 to 30 c.cm. are sufficient, but in the gluteal region as much as 200 or 300 c.cm. may be injected. When the needle is withdrawn the part should be thoroughly massaged, and the massage should be repeated daily until all sign of crepitation has disappeared. In cases of sciatica the injections should be made in the lumbar region and in the course of the nerve down the leg. Dr. Gubb has never used these injections in cases of facial neuralgia, but he sees no reason why they should not be tried in refractory cases.

Sir James Barr's method of treatment for pleural effusions has already been made known to the profession through his publications in the medical journals, but his recent Bradshaw lecture, in which he deals fully with the physics of the pleura and the treatment of pleural effusions, affords a favourable opportunity to again refer to the subject in these columns. The treatment consists essentially in the early removal of the effusion by syphonage and replacing it with sterilised air and a few cubic centimetres of adrenalin solution. Sir James Barr has devised a special apparatus for carrying out these procedures, which he exhibited at the lecture. The principles underlying the method are to prevent an excessive negative pressure caused in the ordinary way by a sudden withdrawal of a large amount of fluid from the pleura, and also to keep the inflamed pleural surfaces apart for a time, and so prevent, as far as possible, pleural adhesions. The immediate effect of a sudden negative pressure in the pleura is dyspnœa and great discomfort to the patient, and this is prevented by using syphonage instead of aspiration, and by introducing the air as soon as a certain proportion of the fluid has been withdrawn. According to the author, the later effects of this negative pressure are hyperæmia and œdema of the lungs and a rapid recurrence of the effusion. These are prevented by the continued pressure of the air in the pleural cavity, the absorption of which allows of a gradual expansion of the collapsed lung. The addition of the adrenalin solutions causes a contraction of the blood-vessels, and diminishes the possibility of a re-accumulation of fluid. In order to hasten the solution and absorption of effused fibrin Sir James Barr suggests the introduction of trypsin into the pleural cavity.

In the latter part of his address Sir James Barr somewhat scathingly criticises the surgeon's methods of operating upon cases of empyema, and accuses them of ignorance of the principles of physics involved in the condition and a lack of ingenuity and intelligence. After this outburst of indignation one naturally looks for a complete subversion of the usual methods adopted by the much-erring surgeon. He is only told, however, that the operation should be carried out under a local anæsthetic or light general anæsthesia, that the incision should be free and in a dependent spot, about the eighth or ninth intercostal space, with removal of a portion of rib if necessary, and that a strip of gauze should be introduced instead of the usual tube. The use of a drainage tube appears to constitute the chief offence of the surgeon in the eyes of Sir James Barr. According to him, everything should be done to secure complete evacuation of pus, and at the same time exclude the entrance of air, so as to re-establish a negative pressure in the pleura and promote the expansion of the lung. He suggests as a dressing a piece of sterile oiled silk to act as a valve and allow the escape of the fluid, but prevent the entry of air. Sir James Barr has given much thought and study to the subject, and his ideas are always worthy of consideration. It is a pity, however, when the expression of personal views is tempered with arrogance and animus towards fellow-workers in the same field.

